# Insights and advances in recurrent vulvovaginal candidiasis

**DOI:** 10.1371/journal.ppat.1011684

**Published:** 2023-11-10

**Authors:** Javier San Juan Galán, Vanessa Poliquin, Aleeza Cara Gerstein

**Affiliations:** 1 Department of Microbiology, Faculty of Science, University of Manitoba, Winnipeg, Manitoba, Canada; 2 Obstetrics, Gynecology and Reproductive Sciences, University of Manitoba, Winnipeg, Manitoba, Canada; 3 Department of Statistics, Faculty of Science, University of Manitoba, Winnipeg, Manitoba, Canada; University of Maryland, Baltimore, UNITED STATES

## Introduction

Vulvovaginal candidiasis (VVC, colloquially referred to as a “yeast infection”) is a vaginal fungal disease that is most common in females between 20 and 50 years old [1]. The colloquial term “yeast” refers to the clinical syndrome and is the technical word for the typical morphology of the causative species. It is commonly stated that 75% of women will have at least 1 VVC episode, with 5% to 10% developing recurrent vulvovaginal candidiasis (“RVVC”) [1]. These statistics may underestimate the true incidence of R/VVC (i.e., referring to both VVC and RVVC). Recent global analyses of RVVC epidemiology estimated that 138 million women are annually affected [2,3]. However, their analyses also highlighted that few to no robust global studies track women for at least 1 year with reliable clinical and microbiological data, and they hypothesize that prevalence and incidence are probably higher than assessed and increasing due to a growing at-risk population [2,3]. R/VVC significantly affects morbidity and quality of life, with women incurring physical pain and elevated levels of depression and anxiety, including between symptomatic episodes [4]. The economic impact on individuals and healthcare systems is also considerable, with global estimated losses of approximately $14 billion due to a decline in productivity [2,4]. It is unclear why most VVC infections are successfully cleared after a single course of drug treatment, while a subset of women incur relapse of symptomatic episodes even during long-term maintenance treatment or after the cessation of therapy ([5] and references within). Whether symptomatic relapse is typically due to the population expansion of persistent vaginal yeast following treatment or reinfection remains unknown [6]. Yeast can be a common member of the vaginal and gastrointestinal microbiome [7] without causing symptoms, further complicating our biological understanding of the interplay between vaginal yeast colonization and pathogenesis [8].

Here, we discuss our current understanding of the etiology and diagnosis methods for R/VVC and highlight exciting recent developments in the treatment landscape.

## Developments in *Candida* taxonomy

**Fig 1:**
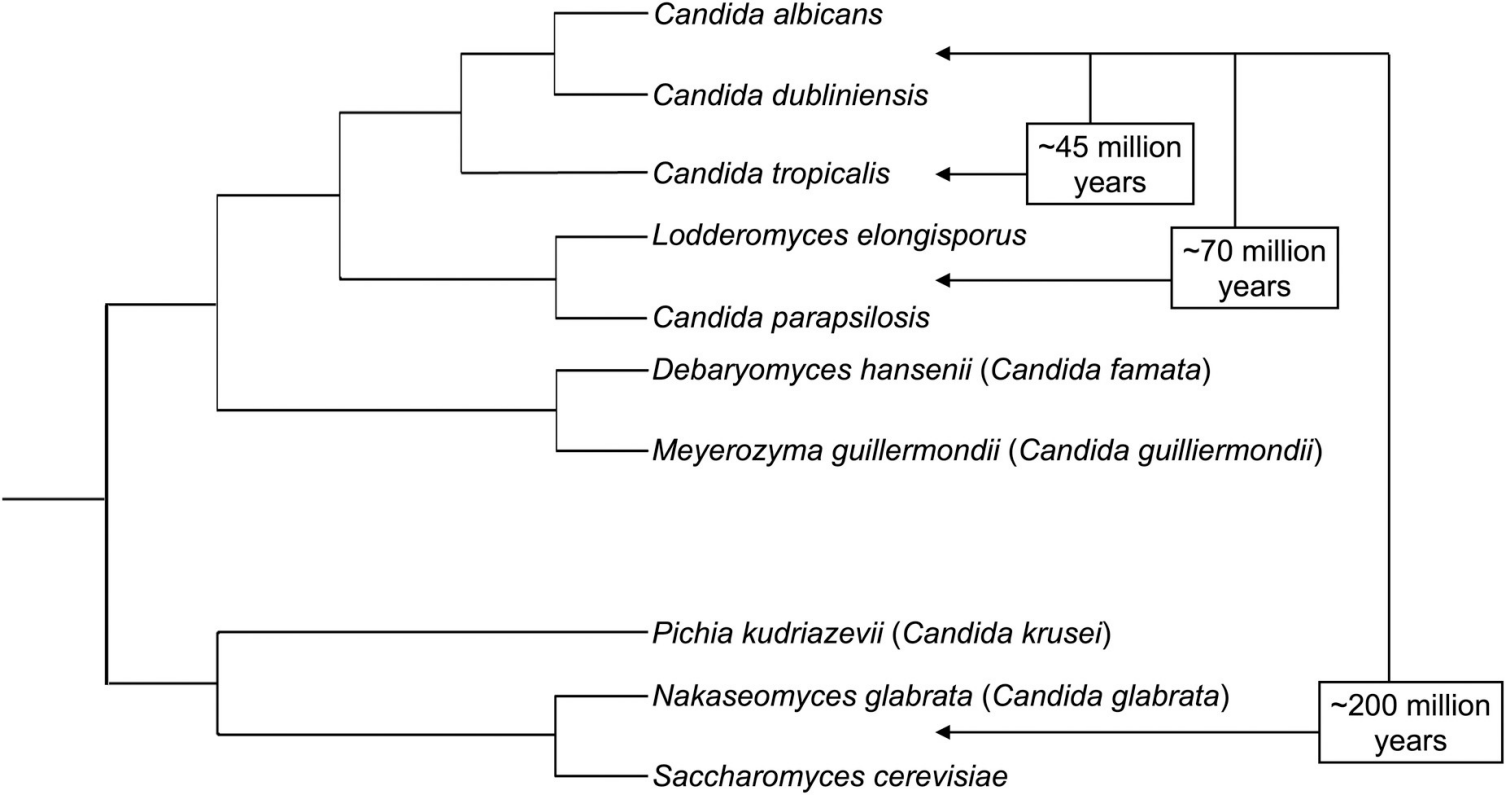
Phylogenetic relationships and approximate evolutionary divergence among VVC yeast species and close relatives. The listed species names reflect the recently proposed nomenclature, with the *Candida* spp. names indicated in brackets. Note that the branch lengths are not proportional to genetic relatedness.

R/VVC is caused by yeasts traditionally classified in the genus *Candida*. However, the genus *Candida* is highly polyphyletic, and several international taxonomic groups have recently endorsed reclassifying 25 species into 13 genera within the order Saccharomycetales [9–11]. The proposed changes include *Nakaseomyces* (*Candida*) *glabrata*, the second most common species implicated in R/VVC (Fig 1). Concerns have been expressed about the impact of name changes on the compatibility with the published literature and the confusion it may generate in clinical practice; however, the name changes are likely far from many clinicians’ radars. Approximately 85% to 95% of R/VVC episodes are caused by *Candida albicans* [6]. Other yeasts cause the remaining cases. We endorse defining these as non-*albicans* clinical yeast (“NACY”) species; the helpful acronym NAC has been commonly used to group species as “non-*albicans Candida*.” Naming is not purely an academic matter. Artificially grouping distantly related species has obscured the substantial evolutionary history differentiating yeast species. This may have impeded progress in understanding differences among infections caused by different species, which should potentially be classified and treated as separate diseases.

## R/VVC diagnosis

VVC can be classified into uncomplicated and complicated cases after an integrated diagnosis with clinical and laboratory evidence [1]. Uncomplicated cases occur as single or sporadic symptomatic episodes, have a mild clinical presentation, and are commonly caused by *C*. *albicans*. In contrast, complicated cases of VVC have more severe symptoms that may require longer treatment, and NACY species isolates are more frequent [1,12]. RVVC is commonly defined as 3 or more symptomatic episodes within a year with remission between episodes, with similar symptomatic presentation as VVC [13–15].

Diagnosis is based on clinical examination, vaginal secretion microscopy, and mycological culture. Observation of filaments (i.e., pseudohyphae or hyphae) can be helpful, though prevalent species like *N*. *glabrata* do not filament [15]. Identifying colonies on mycological culture media is considered the “gold standard” in diagnosis; however, a culture may take 48 to 72 hours to be positive. Chromogenic media can identify some species (e.g., *C*. *albicans*, *C*. *tropicalis*, and *P*. *kudriazevii*) based on colony color, though *N*. *glabrata* and some other NACY species require validation through biochemical or molecular assays [16]. Exciting new approaches are being implemented to improve sensitivity, specificity, and diagnostic speed for the differential diagnosis of VVC from bacterial infections. For example, a metabolome study that examined the lipidomic profile of the vaginal discharge from women with asymptomatic vaginal yeast, women with VVC, and women with cytolytic vaginosis identified biomarkers specific to VVC [17]. Matrix-assisted laser desorption ionization–time of flight mass spectrometry (MALDI-TOF) can identify microorganisms based on their ribosomal protein profiles rapidly and can well differentiate *C*. *albicans* and NACY species [18]. The Aptima CV/TV system, recently approved by the US Food and Drug Administration (FDA), detects the RNA fraction of the ribonuclease P to distinguish *N*. *glabrata* from other VVC-causing species [19]. However, although there is a clinical need for new technological advances to become more widely adopted, it remains a budgetary challenge in the fiscally constrained climate of many places.

## The epidemiological triad in R/VVC: The yeast, the host, and the environment

R/VVC isolates are located throughout the *C*. *albicans* phylogeny. However, they are overrepresented in clade 1 (the most common clade) and comprise almost all of clade 13, which has been proposed to be a closely related yet distinct species, *Candida africana* [20]. Many studies that compared VVC, RVVC, and commensal isolates found similar virulence phenotypes and drug susceptibilities. A very recent study found that VVC isolates compared to commensals exhibited a different interaction with vaginal epithelial cells, triggering a divergent innate immune response [21]. This exciting result points to a potential isolate-level difference with symptomatic consequences.

The host plays a crucial role in R/VVC. The immune system’s tolerance to yeast in the vaginal environment determines the onset of a symptomatic episode. Symptoms result from a non-protective inflammatory response mediated by neutrophils and the innate immune system [22,23]. Strikingly, no clear function has been established for an adaptive response. Rare allelic variation in cell receptors that are correlated with geography and ethnicity has been linked to increased R/VVC susceptibility: dectin-1 and CARD9 polymorphisms are more frequent in African populations compared to other latitudes [24,25], and a US study found a higher frequency of MBL2 variants in black North American women than in white women [26].

*C*. *albicans* populations in the vagina interact with other microbial community members. *Lactobacillus* spp., a prevalent member of the healthy vaginal community [27], may regulate *C*. *albicans* through competition [28]. However, immunocompetent individuals with symptoms can have a similar proportion of lactobacilli in their vaginal microbiota as those found in healthy women [29,30]. Factors like antibiotics that perturb the bacterial microbiome are thought to promote *C*. *albicans* colonization in the vaginal epithelium [31]. R/VVC episodes are highly prevalent in women with frequent antibiotic indications, such as cystic fibrosis [32] and recurrent bacterial vaginosis [33]. Dysregulation of yeast populations in the vaginal mucosa due to treatment for other conditions has also been established, e.g., sodium-glucose cotransporter 2 inhibitors for type 2 diabetes and hormone replacement therapy in postmenopausal women [34,35].

Although some risk factors have been identified (as above), RVVC cases are often classified as idiopathic, and many questions remain about the interaction between yeast, host, and vaginal microbial community. Methods for fungal metagenomics lag behind those for bacteria, yet other fungal species are also in the vagina [7,36]. Microbial populations are currently assessed as proportions rather than absolute numbers, which may conceal important individual differences. Furthermore, very few studies have examined how NACY species interact with the host [31,37], and it is unclear how generalizable the results from *C*. *albicans* are for infection caused by other species.

## Old and new options for R/VVC treatment

Fluconazole has been the frontline treatment for R/VVC since the 1990s due to its safety and efficacy as an oral antifungal. Although still relatively rare, fluconazole resistance can be intrinsic to NACY species isolates. It can also emerge in response to repeated exposure to low-dose fluconazole monotherapy and over-the-counter azoles [38]. Different jurisdictions have different recommendations for when fluconazole fails, though generally nystatin or boric acid are recommended [5,14,39]. Boric acid has been used as a broad-spectrum antimicrobial for over 100 years, with reports of its use in treatment for VVC going back to at least the 1980s [40]. Although its mechanism of action is not fully understood, boric acid impairs hyphal morphogenesis and biofilm formation in *C*. *albicans* and has excellent antifungal activity in *C*. *albicans* and NACY species [41]. Boric acid is also likely to directly influence the vaginal bacterial community in a way that is different than standard antifungal drugs and may provide an additional benefit. Boric acid has proven safe in humans with no severe or moderate effects [42]. Yet, it is only available in Canada through compounding pharmacies or from nonmedical retailers, remains unapproved by the USA FDA [43], is available only in specialty centers in the UK [44], and the EU restricts its use for exceptional cases [13]. Probiotics in combination with conventional treatment have shown a reduction in RVVC symptoms and recurrence rates in small population size studies ([45] and references within). None of the current therapies can prevent recurrence entirely. After a long period without new drugs, promising therapies have recently been approved that may better prevent symptomatic relapse.

In 2022, the FDA officially approved a novel oral azole called oteseconazole (OTZ) for treating R/VVC [46]. OTZ has a higher affinity for the fungal CYP51 than the human orthologue protein, which reduces adverse interactions compared to other azoles [47]. While it has a 10-fold lower minimum inhibitory concentration against *C*. *albicans* and NACY species than fluconazole, OTZ is less effective against azole-resistant mutants that have increased expression of drug efflux pumps [48]. Clinical trials to evaluate the effect of OTZ on women with RVVC reported no severe side effects and that less than 10% of participants experienced recurrence within a year [49,50]. OTZ is contraindicated in pregnant and lactating women due to potential embryo-fetal toxicity [51].

Fungerps are terpenoid inhibitors of β-(1,3)-D-glucan synthase that was approved by the FDA in 2021 and are beginning to be used to treat R/VVC [52]. The first oral candidate, ibrexafungerp (IBX), has shown excellent antifungal activity against a broad spectrum of yeast species, including echinocandin and fluconazole-resistant isolates [53]. Clinical trials of women with RVVC treated with IBX compared to a placebo have reported higher rates of clinical cure and negative yeast cultures, with approximately 10% fewer cases with recurrence in ≥6 months [54]. IBX is currently bound to patent regulations that limit its prescription outside the US, and like OTZ, is limited to nonreproductive potential females due to the risk of fetal harm [55].

In addition to the newly approved antifungals, an immunotherapeutic candidate vaccine, NDV-3A, targets the adhesin agglutinin-like sequence 3 (Als3) of *C*. *albicans*, has also been developed [56]. NDV-3A is a recombinant protein of Als3 that activates B and T-cell response and enhances mucosal immunity without affecting the immune response against *C*. *albicans* in other anatomical sites [56]. In a controlled trial, the proportion of vaccinated participants with no relapse episodes during the study period was double the non-vaccinated population.

## Conclusions and future directions

The research community is currently seeing significant advances in understanding and treating R/VVC. The current landscape for diagnosis seems promising, with highly sensitive and specific technologies that would allow the monitoring of the vaginal environment and predictions of recurrent symptomatic episodes. Novel therapeutic alternatives, waiting for global commercialization, have also emerged for R/VVC, showing effective outcomes. Our understanding of the ecological relationships in the vaginal community is also expanding through the integration and technological improvements in metagenomics, which will provide key evidence in the pathogenesis and etiology of the disease. However, advocacy is sorely needed for women’s health to be a priority for funders and decision-makers to make this a reality.
